# Preparation and Characterization of a Binary Form-Stable Composite Phase-Change Material Based on n-Decanol and n-Octanoic Acid

**DOI:** 10.3390/ma19183983

**Published:** 2026-09-19

**Authors:** Guanyu Zhu, Hongde Zhen, Nan Wang, Rumeng Zhang, Biao Zhou, Bin Guo

**Affiliations:** College of Water Conservancy and Civil Engineering, Shandong Agricultural University, Tai’an 271018, China; 2024121392@sdau.edu.cn (G.Z.); 2024121391@sdau.edu.cn (H.Z.); 2024110744@sdau.edu.cn (N.W.); 2024110746@sdau.edu.cn (R.Z.); 2023121316@sdau.edu.cn (B.Z.)

**Keywords:** composite phase-change materials, latent heat energy storage, n-decanol, n-octanoic acid

## Abstract

Phase-change energy storage materials have the potential to improve the durability of hydraulic concrete; however, phase-change materials suitable for dam engineering within the 0–5 °C phase-transition range remain limited. In this study, a binary phase-change material was prepared by melt blending n-decanol (DA) and n-octanoic acid (CA). Expanded vermiculite (EV), with a layered porous structure, was selected as the form-stabilizing support matrix. A thermally stable DA-CA/EV composite phase-change material was then prepared by melt impregnation and adsorption, and its thermal properties, shape-stabilization performance, and thermal reliability were systematically characterized. The results indicate that the optimal binary composition is 35 wt.% DA and 65 wt.% CA, with a solidification phase-transition temperature of 2.5 °C and a latent heat of 163 kJ/kg. Moreover, the use of 100-mesh EV as the supporting matrix effectively prevents leakage. The optimal thermal conductivity is achieved when approximately 50 wt.% PCM is adsorbed while a high bulk density is maintained. Owing to its favorable thermal performance and structural stability, the prepared PCM shows promise for application in hydraulic concrete.

## 1. Introduction

Water conservancy projects are a critical component of national infrastructure. Owing to their large scale, technical complexity, and high investment requirements, major hydraulic engineering structures impose particularly stringent durability demands on hydraulic concrete. This requirement is especially important for structures in severely cold regions, where freeze–thaw damage is pronounced [1,2,3]. Current studies indicate that epoxy resins can effectively reduce liquid penetration into concrete [4]. Kostrzewski et al. [5] investigated water-dilutable epoxy resins as internal protective agents for concrete and found that an appropriate resin dosage can limit the ingress of aggressive ions and mitigate sulfate attack while maintaining overall material performance. Guo et al. [6] developed a shrinkage-compensating concrete containing a double expansive admixture and evaluated its frost resistance through rapid freeze–thaw cycling. After 150 cycles, corresponding to the stable-damage stage, the concrete with a U-type expansive agent:MgO expansive agent ratio of 2:1 exhibited the best frost resistance, with RDME increased by 17.35% and MLR reduced by 25.1% relative to concrete without expansive agents. Other studies have shown that cement mortar incorporating fly ash and limestone powder and cured at 5 °C can effectively enhance concrete freeze–thaw resistance [7].

Several researchers have proposed that phase-change materials (PCMs) can delay the freezing of pore water under sub-zero temperatures by releasing latent heat, thereby stabilizing the pore-size distribution and improving the frost resistance of concrete [8]. However, experimental implementation of this approach in concrete remains limited. With increasing energy demand driven by economic development, phase-change energy storage technology, which absorbs or releases energy during material phase transitions, has attracted extensive attention [9,10]. It has been widely applied in building energy conservation [11,12], solar energy utilization [13], air-conditioning systems [14], and refrigerated food transportation [15].

PCMs for energy storage are generally classified as inorganic or organic materials [16]. Inorganic PCMs often suffer from phase separation and significant supercooling, which adversely affect their cold-storage performance [17,18]. In contrast, organic PCMs generally exhibit favorable thermal stability and limited supercooling [19]. Nevertheless, single organic PCMs often cannot fully satisfy practical application requirements. Based on the eutectic-mixture principle, multiple PCMs are therefore commonly combined to adjust phase-transition temperatures and improve latent heat storage capacity [20,21]. Zhang et al. [22] prepared a binary composite PCM using decanoic acid and myristic acid and determined a eutectic mass ratio of 7:3, with a solidification phase-transition temperature of 26 °C. Zuo et al. [23] prepared a decanoic acid–octadecanol binary PCM with an optimal mass ratio of 85.15:14.86, a solidification phase-transition temperature of 26.48 °C, and a latent heat of 181.06 J/g; the material exhibited good thermal stability and practical potential for building energy conservation. Zhou et al. [24] prepared paraffin–lauric acid and paraffin–myristic acid composites with solidification phase-transition temperatures of 36.88 and 42.17 °C and latent heats of 174.55 and 184.53 J/g, respectively, demonstrating effective energy storage and utilization. Ayaz et al. [25] investigated a caprylic acid–stearyl alcohol mixture; differential scanning calorimetry showed onset melting/solidification temperatures of 11.4/11.8 °C and fusion/freezing latent heats of 154.4/150.5 J/g, indicating excellent thermal reliability and stability.

Because PCMs become fluid in their molten state, leakage can occur during solid–liquid phase transition, which limits their practical application [26]. Although microencapsulation can effectively address leakage, it involves complex processing and a relatively high cost [27]. In contrast, physical adsorption of molten PCM into high-surface-area porous matrices through capillary forces and surface tension provides a simpler and more cost-effective route for leakage prevention [28]. Expanded vermiculite (EV) is an excellent adsorbent owing to its high specific surface area, high porosity, and strong adsorption capacity [29,30]. Yang et al. [31] modified EV through organic intercalation and prepared a series of form-stable paraffin/EV PCMs by physical impregnation, demonstrating good chemical stability. Liu et al. [32] prepared form-stable lauric–myristic acid/modified EV composite PCMs with enhanced heat-transfer performance, showing considerable potential as energy-storage materials for energy-efficient buildings.

At present, PCMs suitable for dam-related applications within the 0–5 °C temperature range remain scarce. To address this limitation, this study designed and prepared an n-decanol/n-octanoic acid binary eutectic PCM. To mitigate liquid leakage during solid–liquid phase transition, EV with a layered porous structure was selected as the form-stabilizing matrix. Form-stable composite PCMs were prepared by melt impregnation, and their shape-stabilization performance, thermal properties, and cycling stability were systematically characterized. The results provide a scientific basis and a feasible material option for applying PCMs in dam engineering.

## 2. Materials and Methods

### 2.1. Materials and Equipment

n-Decanol (DA, C_10_H_22_O, 98%) and n-octanoic acid (CA, C_8_H_16_O_2_, analytical reagent grade, 99%) were purchased from Shanghai Macklin Biochemical Co., Ltd. (Shanghai, China). Expanded vermiculite (EV, 100 mesh) was supplied by Shijiazhuang Yuxin Building Materials Co., Ltd. (Shijiazhuang, China). All other analytical-grade reagents were purchased from Sinopharm Chemical Reagent Co., Ltd. (Shanghai, China). All raw materials were used as received without further purification or treatment.

The main instruments used in this study included a heat-collecting constant-temperature magnetic stirrer DF-101S (Shanghai, China), a thermocouple temperature recorder K-612C (Suzhou, China), a scanning electron microscope gold sputtering system ETD-900M (Brno, Czech Republic), a scanning electron microscope Axia ChemiSEM (Brno, Czech Republic), a differential scanning calorimeter DSC 3500 Sirius (Selb, Germany), a Fourier-transform infrared spectrometer iS5 (Madison, WI, USA), a thermogravimetric analyzer STA200 (Hitachi High-Tech), and a multifunctional rapid thermal conductivity tester DRE-III (Xiangtan, China).

### 2.2. Preparation of Form-Stable Composite Phase-Change Materials

**Figure 1:**
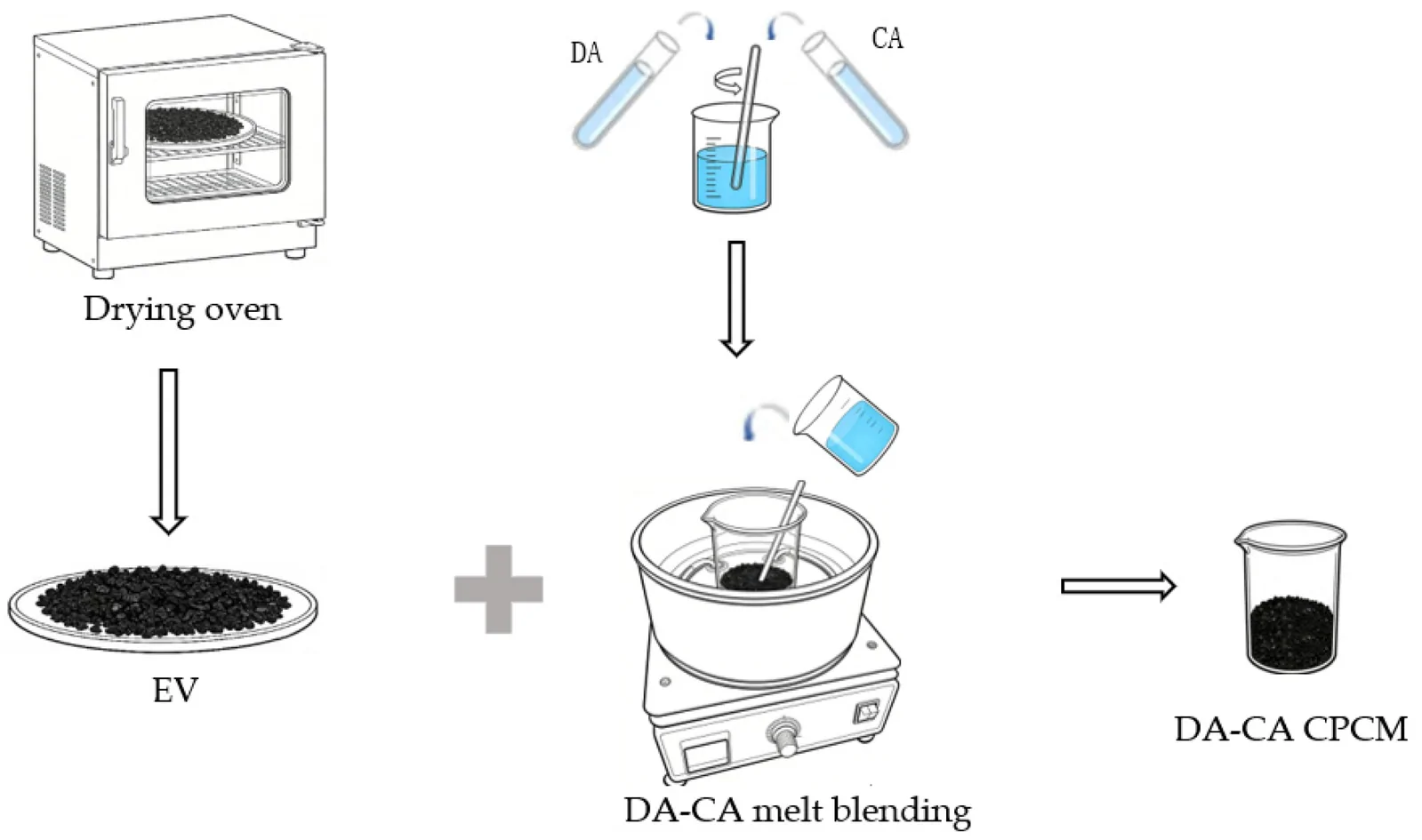
Flowchart of the DA-CA/EV preparation process.

The preparation procedure for the EV-based composite PCM is illustrated in Figure 1. Predetermined amounts of DA and CA were weighed and mixed in a beaker. The beaker was placed on a constant-temperature magnetic stirrer, and the mixture was heated and continuously stirred for 30 min. EV was dried in an oven for 60 min to remove moisture. Subsequently, the molten DA-CA mixture was added to the dried EV and stirred at 60 °C for 30 min to ensure sufficient adsorption.

### 2.3. Experimental Characterization of Phase-Change Materials

#### 2.3.1. Hot-Melt Step-Cooling Test

A beaker containing the binary PCM was placed in a constant-temperature water bath at 80 °C. After heating for 20 min, the beaker was removed, and a thermocouple was inserted into the center of the sample without contacting the beaker wall or bottom. The sample was then rapidly transferred to a −10 °C freezer until complete crystallization, and the temperature was recorded every 5 s.

#### 2.3.2. Encapsulation Efficiency Test

The diffusion-bleeding ring method was used to quantitatively evaluate the leakage behavior of the DA-CA/EV composite PCM. A measured amount of composite PCM particles was uniformly distributed within the designated test area and left at room temperature for 24 h. The filter paper surface was then examined. The appearance of an oil-stain ring outside the test area, caused by migration of the molten PCM, was taken as evidence of leakage.

#### 2.3.3. SEM Analysis

The sample surface was observed using scanning electron microscopy (SEM). Signals generated by the interaction between the incident electrons and the sample were detected to obtain information on the microstructure, composition, and morphology of the sample. The magnification range was 5× to 1,000,000×.

#### 2.3.4. Differential Scanning Calorimetry Testing

The phase-transition temperature and latent heat of the binary PCM were determined by differential scanning calorimetry (DSC). The test conditions were as follows: sample mass, 12–15 mg; temperature range, −40 to 80 °C; heating/cooling rate, 5 °C/min; nitrogen atmosphere; and nitrogen flow rate, 50 mL/min.

#### 2.3.5. FT-IR Analysis

The chemical structures of the samples were characterized by Fourier-transform infrared (FT-IR) spectroscopy. Spectra were recorded over the range of 4000–500 cm^−1^.

#### 2.3.6. Thermal Stability Analysis

Thermogravimetric analysis (TGA) was used to evaluate the thermal stability of the individual raw materials and the prepared composite samples. The tests were conducted under a nitrogen atmosphere at a heating rate of 10 °C/min over a temperature range of 30–600 °C.

### 2.4. Theoretical Prediction of Phase-Transition Temperature and Latent Heat

In this study, n-decanol (molecular weight: 158.28; melting point: approximately 7 °C) and n-octanoic acid (molecular weight: 144.21; melting point: approximately 16 °C) were used. To obtain a binary composite with an appropriate phase-transition temperature and latent heat, the optimal composition was determined through both theoretical calculation and experimental measurement.

According to the theoretical prediction of melting points and fusion enthalpies for eutectic PCMs, the theoretical phase-transition temperature and latent heat of organic eutectic mixtures can be calculated using Equations (1) and (2) [33].

(1) $$T_{m}={\left(\frac{1}{T_{i}}-\frac{RlnX_{i}}{H_{i}}\right)}^{-1}$$

(2) $$H_{m}=T_{m}\left(\frac{X_{A}H_{A}}{T_{A}}+\frac{X_{B}H_{B}}{T_{B}}\right)$$

In these equations, *T_i_* is the melting temperature of component *i* (K); *T_m_* is the melting temperature of the mixture (K); *H_i_* is the latent heat of fusion of component *i* (J/mol); *H_m_* is the latent heat of fusion of the mixture (J/mol); *X_i_* is the molar fraction of component *i* in the mixture, where *X_A_* + *X_B_* = 1; and *R* is the universal gas constant, 8.31 J/(mol·K).

## 3. Results and Discussion

### 3.1. Determination of the Eutectic Point of Binary Phase-Change Materials

#### 3.1.1. Theoretical Prediction of the Eutectic Point

**Figure 2:**
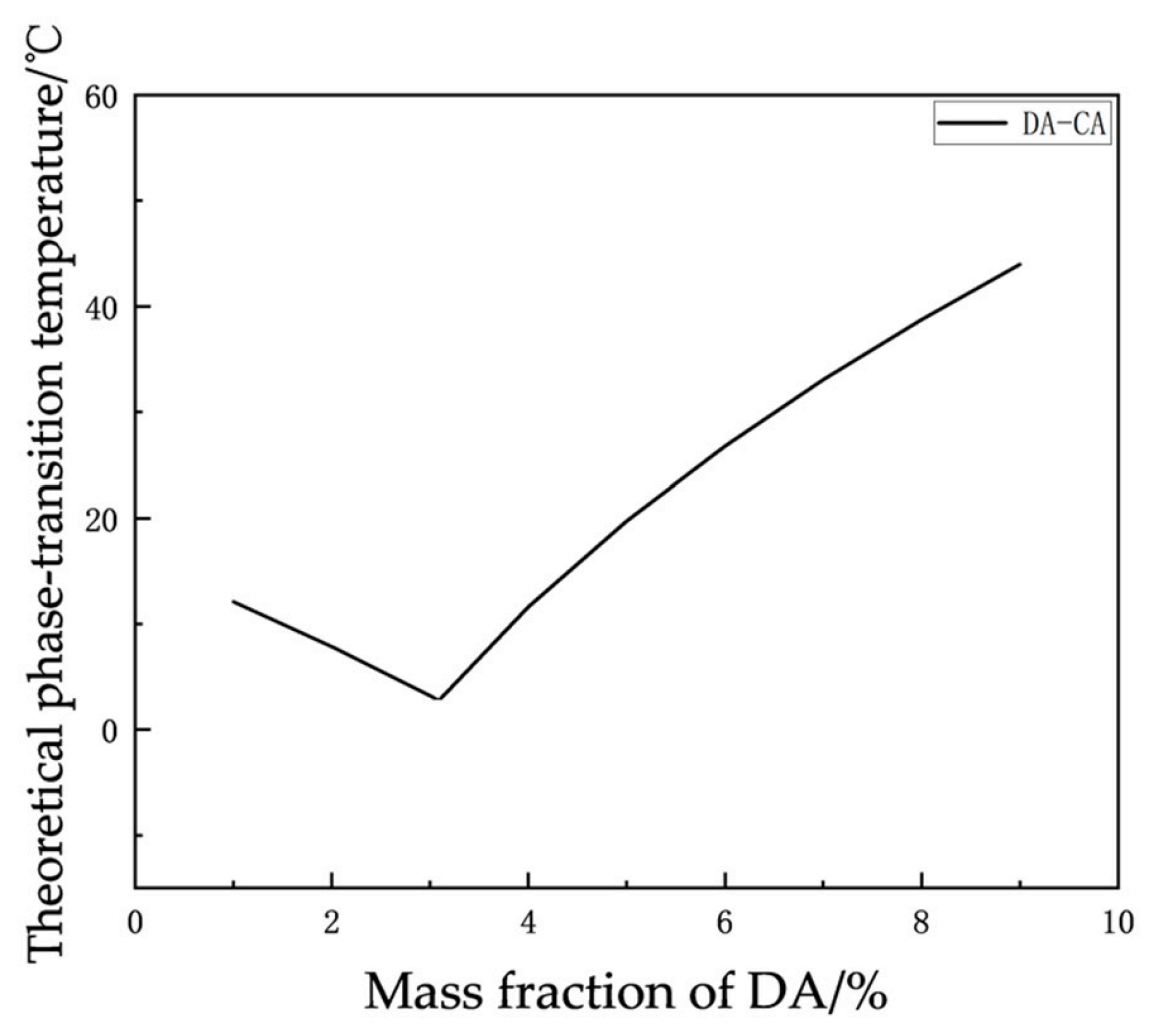
Binary eutectic phase diagram.

The phase-transition temperature and latent heat of fusion of the mixture were calculated using Equations (1) and (2), and the results are shown in Figure 2. The theoretical molar ratio of the DA-CA binary eutectic mixture was calculated as 31:69. The corresponding theoretical solidification temperature and latent heat of solidification were 2.7 °C and 19,706.92 J/mol, respectively.

#### 3.1.2. Step-Cooling Curve Analysis

**Figure 3:**
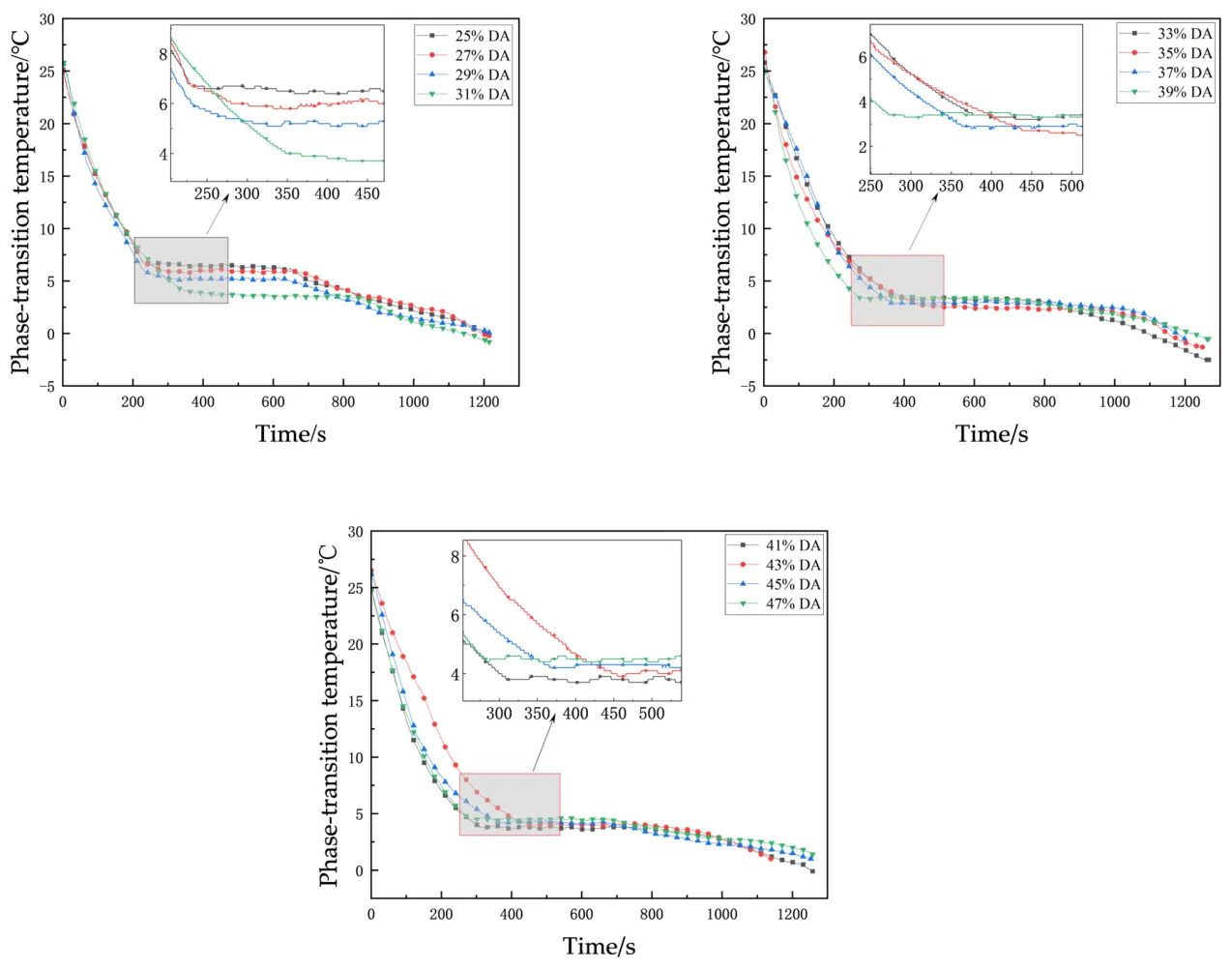
Step-cooling curves of DA-CA at different mass ratios.

To obtain more accurate phase-transition temperatures and latent heat values for the binary PCM, experimental comparison and validation were required. Twelve composite PCM formulations near the theoretical molar ratio were selected. Specifically, samples with DA mass fractions ranging from 25% to 47% were prepared and tested, as shown in Figure 3.

As shown in Figure 3, when the DA mass fractions were 33%, 35%, and 37%, the corresponding solidification temperatures of DA-CA were 3.3, 2.5, and 3.4 °C, respectively. Therefore, the minimum eutectic temperature of DA-CA was determined to be 2.5 °C, and the optimal mass ratio was 35:65.

**Figure 4:**
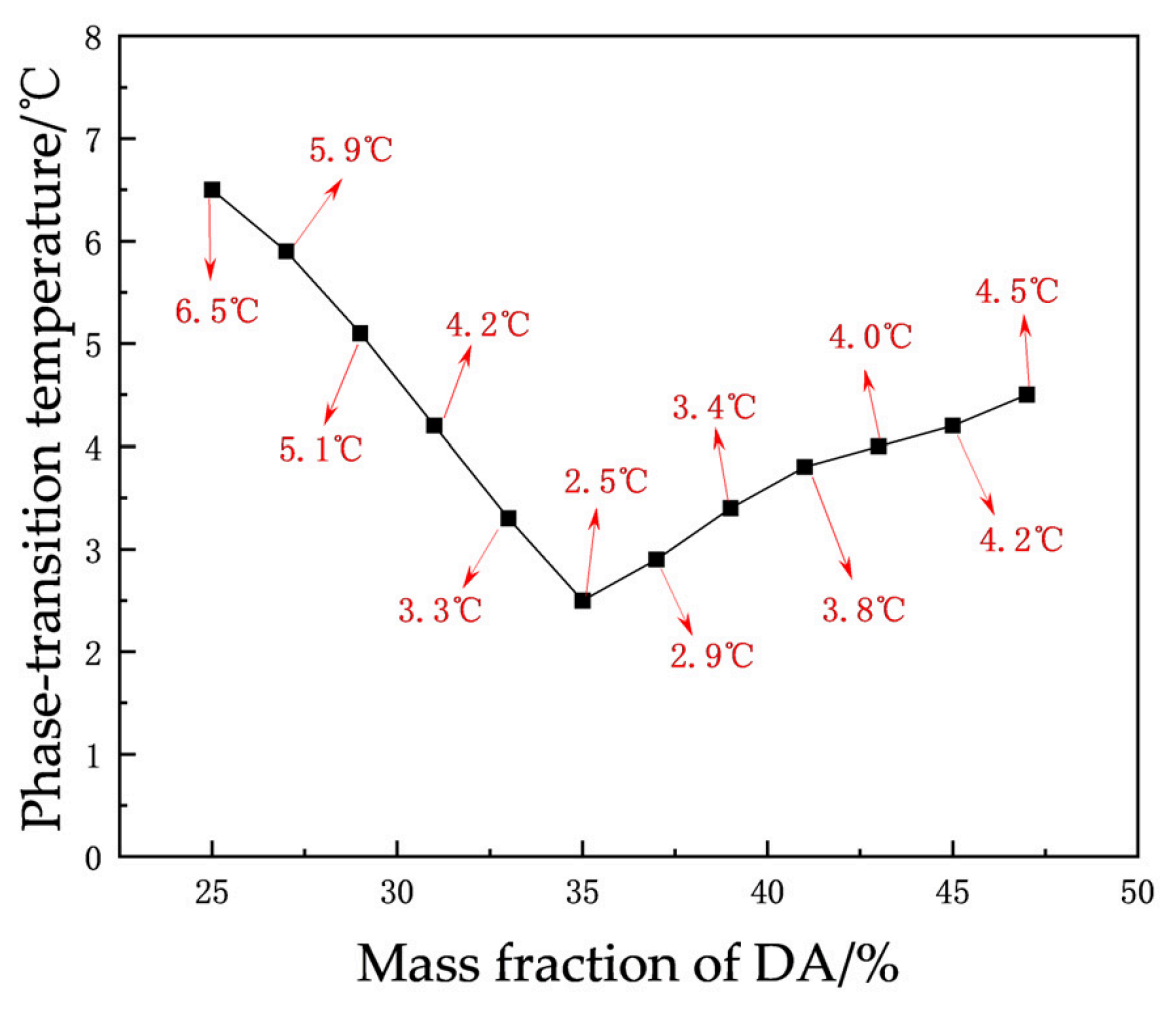
Local phase diagram of DA-CA.

As shown in Figure 4, as the concentration of n-decanol in the system gradually decreased, the phase-transition temperature of the binary PCM generally declined. Specifically, the solidification phase-transition temperature decreased from 6.5 to 2.5 °C and subsequently increased to 4.5 °C. The step-cooling tests therefore confirmed that the minimum eutectic point of the DA-CA binary PCM was 2.5 °C.

#### 3.1.3. DSC Analysis

**Figure 5:**
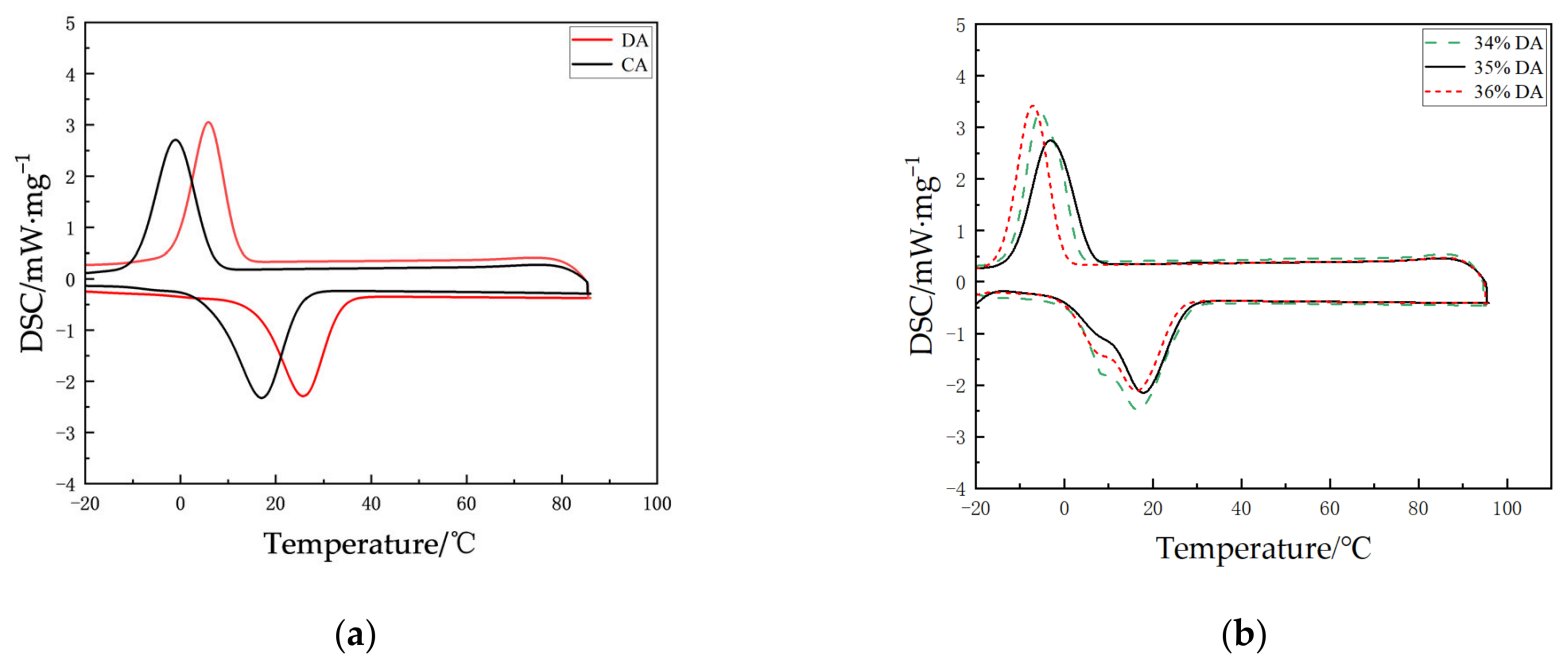
DSC curves: (**a**) DA and CA; (**b**) DA-CA at different mass ratios.

Figure 5a presents the DSC results for n-decanol and n-octanoic acid, which show solidification phase-transition temperatures of 6.8 and 16.5 °C, respectively. Figure 5b shows the DSC curves obtained for compositions near the lowest phase-transition temperature identified from the step-cooling tests. When the mass fraction of DA reached 35%, the solidification and melting temperatures of the DA-CA system reached their respective minimum values of 2.45 °C and 23.08 °C, with a supercooling of 20.63 °C; the latent heat of solidification was 163 J/g, and the latent heat of fusion was 169.6 J/g.

### 3.2. Encapsulation Efficiency of Expanded Vermiculite

#### 3.2.1. Adsorption Efficiency

Current research often employs diatomaceous earth, expanded perlite, and mesoporous silica to adsorb phase-change materials; however, when these matrix materials adsorb phase-change materials, some of the phase-change material remains on the surface of the matrix. When subsequently added to concrete, this residual material reacts with the cement, thereby affecting the material’s performance. In contrast, when expanded vermiculite adsorbs phase-change materials, it forms a microsphere that encapsulates the phase-change material, preventing it from coming into contact with other materials. EV with particle sizes ranging from 40 to 150 mesh was selected and thoroughly mixed with the PCM to promote adsorption. Table 1 summarizes the adsorption efficiency of EV with different particle sizes for the PCM.

As shown in Table 1, EV with particle sizes of 80–150 mesh formed shaped composite PCM particles with diameters of 3–10 mm after PCM adsorption, whereas 40-mesh EV failed to form particles under the same conditions. This result is likely attributable to the larger particle size and relatively smaller specific surface area of 40-mesh EV, which limits the amount of PCM that can be adsorbed per unit mass of vermiculite. In addition, the adsorption rate increased slightly as the EV particle size decreased, primarily because smaller particles improved the wetting efficiency and provided more favorable transport pathways for PCM infiltration into the pores.

#### 3.2.2. Leakage Analysis

To investigate the effect of EV particle size on encapsulation stability, samples with three particle sizes—80, 100, and 150 mesh—were selected. Two parallel tests were conducted for each particle size, yielding six samples in total. The diffusion-bleeding ring method [34] was used to calculate the leakage percentage and evaluate leakage severity. Leakage was quantified as the ratio of the leakage area to the original test area, as calculated using Equation (3). According to the leakage-stability evaluation criteria in Table 2, the calculated leakage percentage was compared with the specified thresholds to evaluate PCM leakage and determine material stability.

(3) $$\varphi =\frac{(D_{max}+D_{min})/2-D}{D}\times 100\%$$

In Equation (3), *D* denotes the diameter of the defined test area (mm), while *D_max_* and *D_min_* denote the maximum and minimum diameters of the PCM extrusion area (mm), respectively.

**Figure 6:**
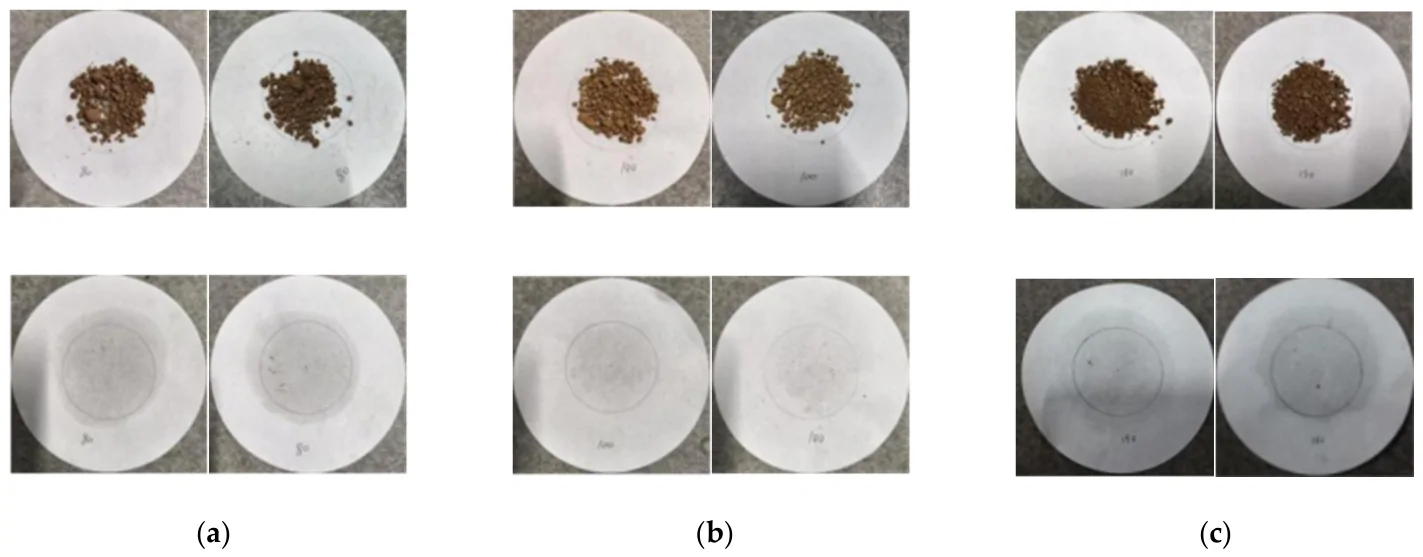
Effect of EV particle size on leakage: (**a**) 80 mesh; (**b**) 100 mesh; (**c**) 150 mesh.

As shown in Figure 6, the encapsulation performance of EV varied markedly with particle size. For the 80- and 150-mesh samples, a large amount of DA-CA adhered to the surface, accompanied by severe liquid-phase leakage, and the leakage area clearly extended beyond the designated test area. The leakage rates calculated by the diffusion-bleeding ring method were 32.34% and 30.92%, respectively, indicating that these composites were unstable. In contrast, the 100-mesh EV sample exhibited highly stable encapsulation performance, with no obvious flaky leakage within the test area, indicating that this particle size provided the best encapsulation effect for the PCM.

### 3.3. Morphological Characterization

**Figure 7:**
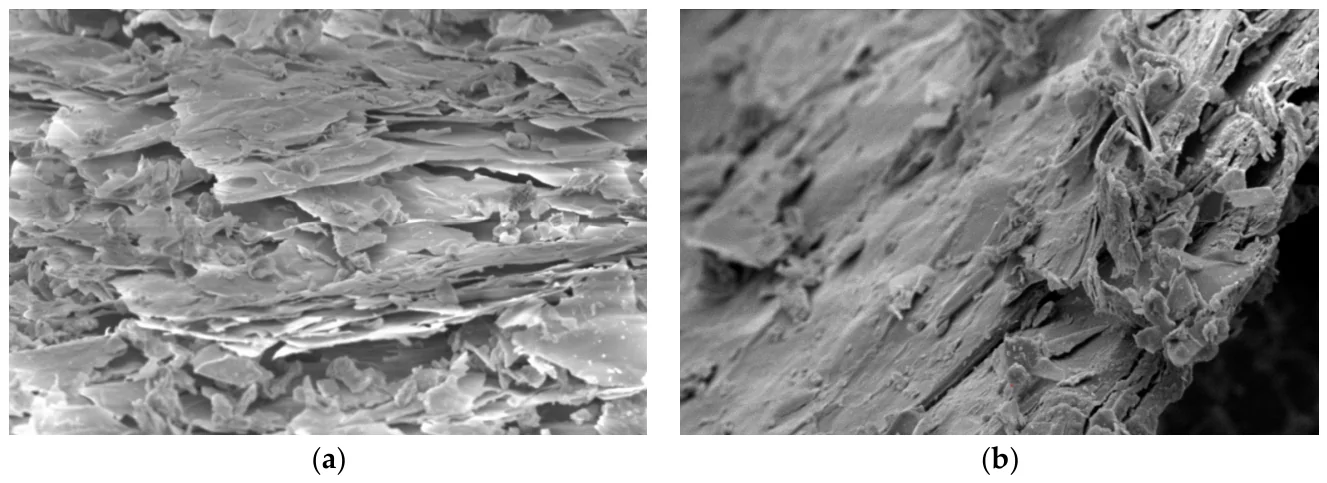
Microscopic morphology: (**a**) EV (×8000); (**b**) DA-CA/EV (×8000).

Figure 7 shows the microstructures of EV and DA-CA/EV. As shown in Figure 7a, EV exhibits a typical layered porous structure, with numerous closely packed pores between the layers. Its high specific surface area and porosity facilitate DA-CA adsorption. Figure 7b shows the morphology of DA-CA/EV after vacuum impregnation. The EV pore size was significantly reduced, and the interlayer spaces were filled with PCM, resulting in a thickened flake-like structure. This morphology is mainly attributed to the combined effects of capillary forces and surface tension within the EV matrix, which stabilize PCM adsorption in the pores and thereby form a structured composite phase-change thermal storage system.

### 3.4. DSC Analysis of DA-CA/EV

**Figure 8:**
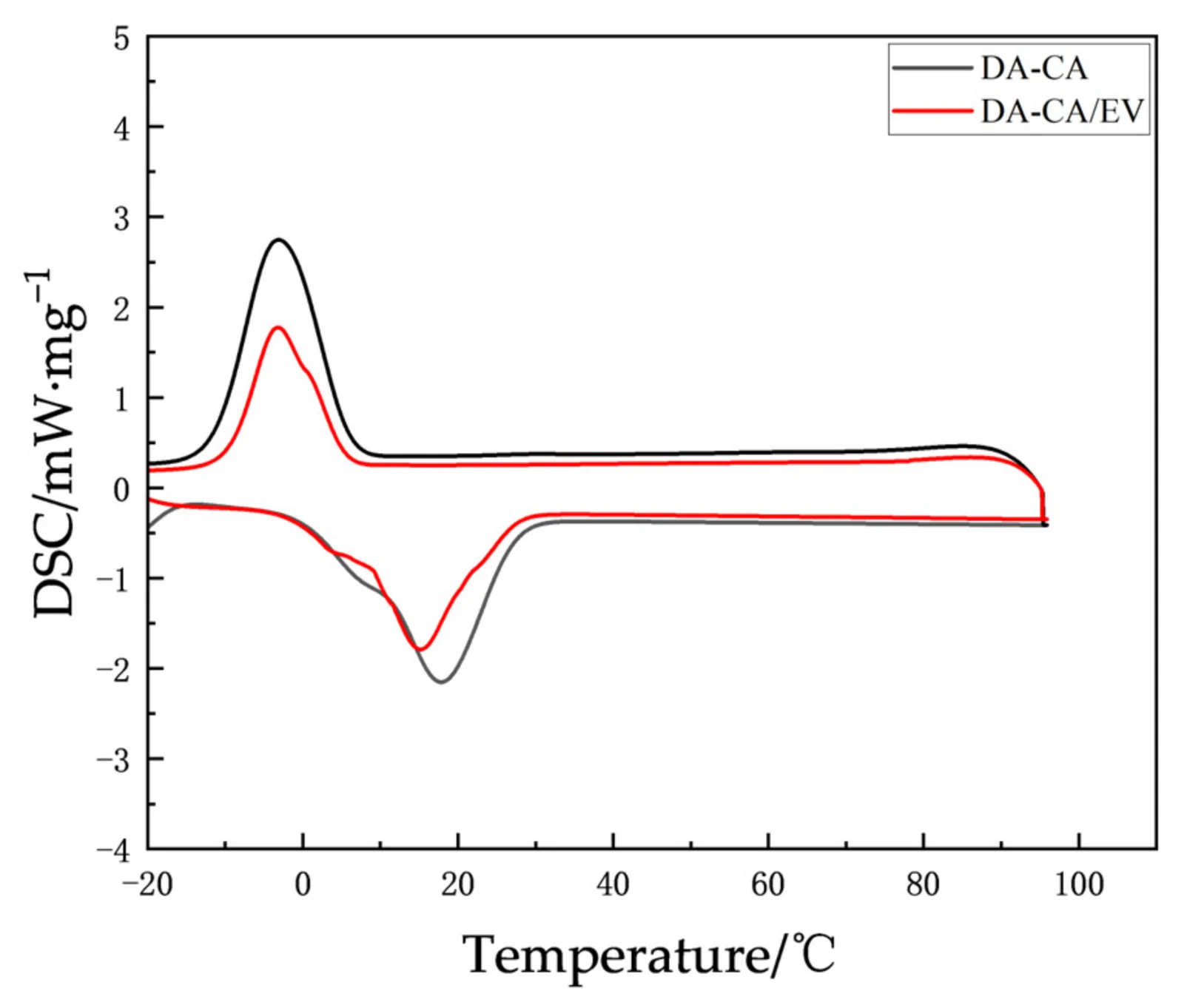
DSC curves of DA-CA and DA-CA/EV.

As shown in Figure 8, the solidification temperature of the DA-CA/EV composite PCM was 2.37 °C, which was only 0.13 °C lower than that of DA-CA. This slight decrease may be attributed to the abundant pores in EV; after the PCM was adsorbed into these pores, interfacial effects slightly reduced the phase-transition temperature. The supercooling was 14.76 °C, a 28.45% decrease compared to DA-CA. Shape-stabilized composite phase-change materials were fabricated by adsorbing the phase-change matrix onto the porous skeleton of expanded vermiculite. The supercooling degree of the composite was reduced to a certain extent, yet severe supercooling still remained in the system, which requires further optimization. This can be attributed to the fact that both n-octanoic acid and n-decanol are straight-chain polar organic compounds, whose crystallization requires the ordered arrangement of molecular chain segments and the reconstruction of hydrogen-bonding networks. As a result, their homogeneous nucleation rates are relatively low, and both compounds inherently exhibit pronounced supercooling during phase transition. The latent heats of fusion and solidification of DA-CA/EV were 120.8 and 113 J/g, respectively. The reduction in latent heat compared with DA-CA is mainly due to the presence of EV, which decreases the mass fraction of active PCM in the composite.

As shown in Table 3, most low-temperature PCMs from previous studies with a phase-transition temperature below 5 °C had low latent heat values. The PCM fabricated herein delivers a superior latent heat and thus better meets the requirements of engineering practices. However, expanded vermiculite (EV) shows relatively weak adsorption performance.

### 3.5. Thermal Cycling Analysis

Table 4 summarizes the mass variation of the specimens after 300 melting–freezing thermal cycles. As shown in Table 4, the mass loss rate was 0.87% after 100 cycles and increased to 1.02% after 200 cycles. Further cycling beyond 200 cycles caused negligible additional mass variation. No oil stains were observed on the filter papers throughout the cyclic test, indicating that the PCM component did not leak. These results confirm that the prepared composite PCM has favorable adsorption capacity and excellent thermal stability.

**Figure 9:**
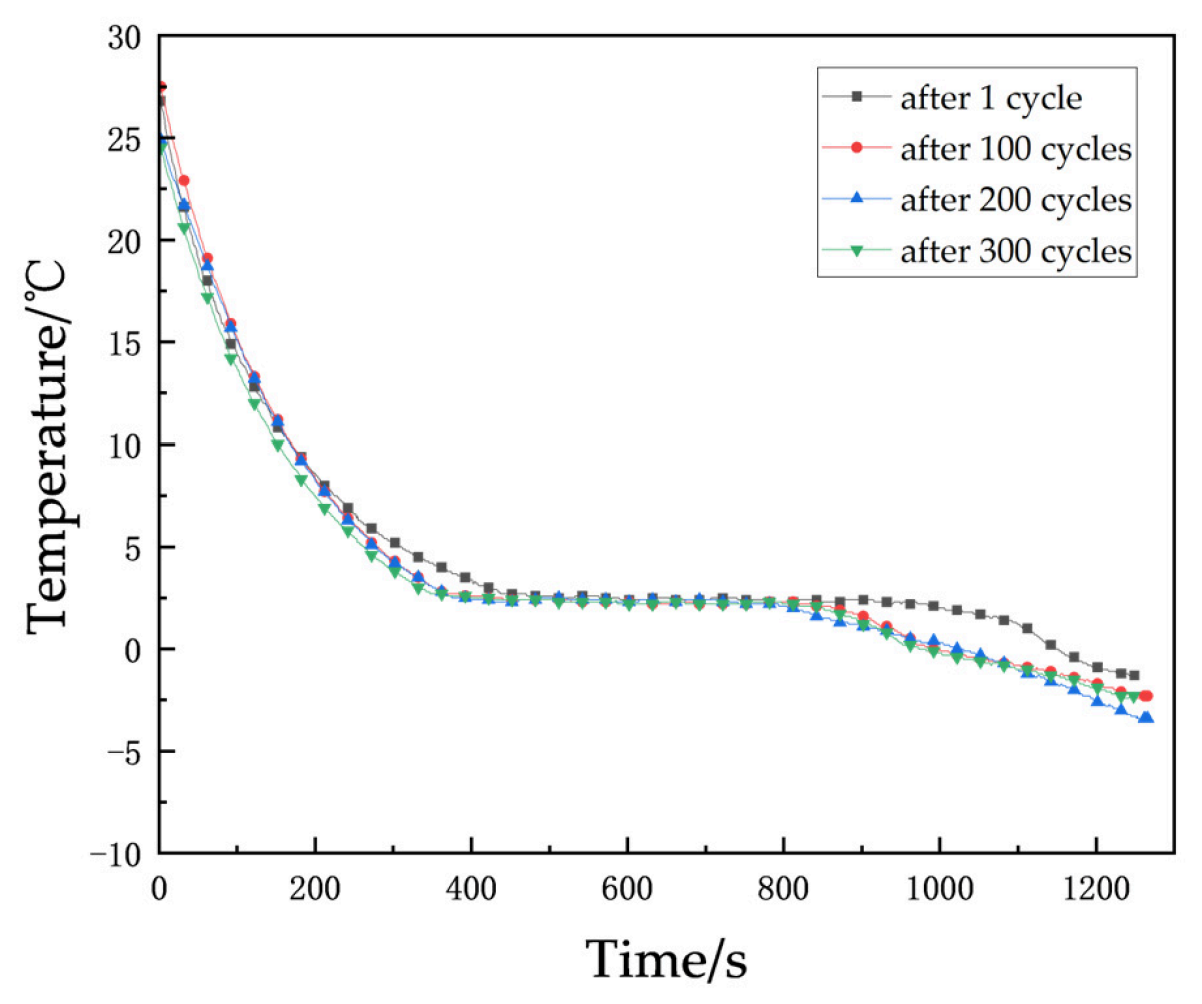
Step-cooling curves of DA-CA/EV after freeze–thaw cycling.

Figure 9 presents the temperature curves of the DA-CA/EV composite PCM after freeze–thaw cycling. After 1, 100, 200, and 300 cycles, the solidification phase-transition temperature remained within a narrow range of 2.2–2.3 °C. This near-constant phase-transition temperature after repeated cycling confirms the excellent thermal reliability of the DA-CA/EV composite PCM.

### 3.6. Structural Characterization

**Figure 10:**
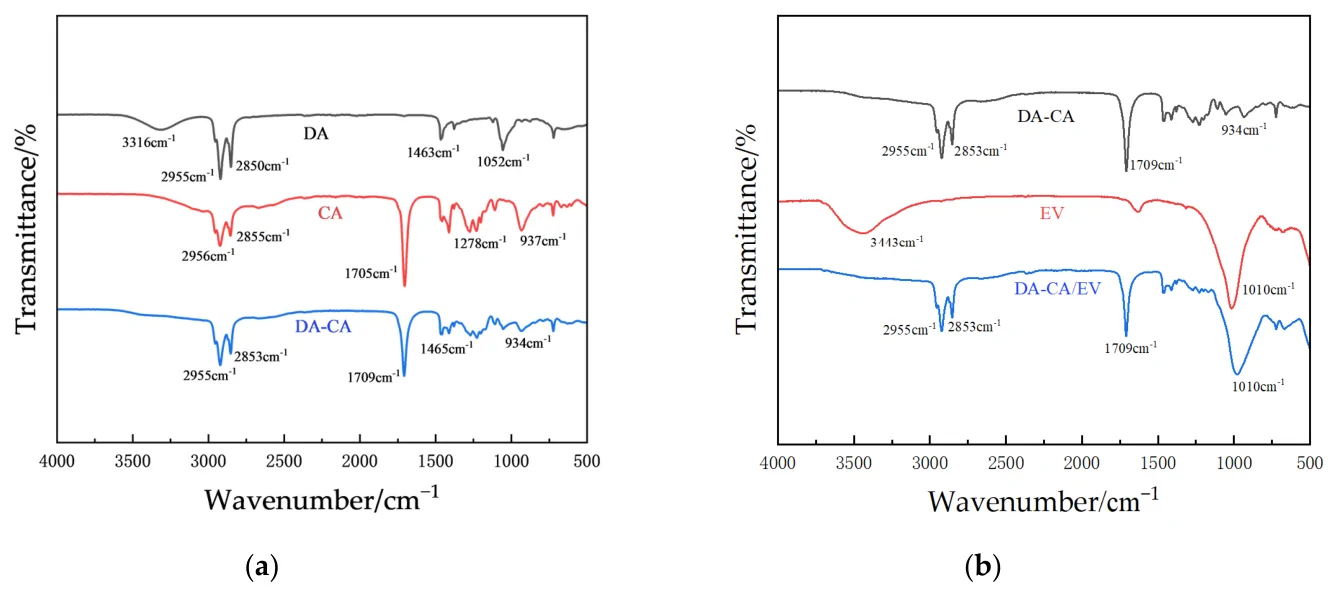
FT-IR spectra: (**a**) DA, CA, and DA-CA; (**b**) DA-CA, EV, and DA-CA/EV.

As shown in Figure 10a, DA exhibits a stretching vibration peak of hydroxyl groups (–OH) at 3316 cm^−1^. The absorption bands at 2955 cm^−1^ and 2850 cm^−1^ correspond to the C–H stretching vibrations of alkyl groups, while the band at 1463 cm^−1^ is assigned to the C–H bending vibration of alkyl groups. The characteristic peak located at 1052 cm^−1^ originates from the C–O stretching vibration.

For CA, the peaks at 2956 cm^−1^ and 2855 cm^−1^ are attributed to the stretching vibrations of –CH_3_ and –CH_2_ groups, respectively. The characteristic absorption peak at 1705 cm^−1^ represents the C=O stretching of carboxyl groups, the band at 1278 cm^−1^ is ascribed to C–O stretching vibration, and the peak at 934 cm^−1^ arises from the bending vibration of –OH groups.

DA-CA exhibits characteristic peaks at 2955, 2853, 1709, 1465, 1275, 1052, and 934 cm^−1^. Compared with the peak positions of DA and CA, no new absorption peaks appeared, indicating that DA and CA were physically blended rather than chemically reacted.

As shown in Figure 10b, the characteristic peak of EV at 3443 cm^−1^ is assigned to –OH stretching vibration, while the peak at 1010 cm^−1^ corresponds to Si–O–Si stretching vibration.

The composite DA-CA/EV shows characteristic peaks at 2955 cm^−1^, 2853 cm^−1^, 1709 cm^−1^, 1465 cm^−1^ and 1010 cm^−1^, which are basically consistent with those of pure DA-CA. No obvious shift is observed for the C–H peaks of alkyl groups and C=O carbonyl peaks. This result demonstrates that no chemical reaction occurs between DA-CA and EV, and the two components are merely physically blended.

### 3.7. TG Analysis

**Figure 11:**
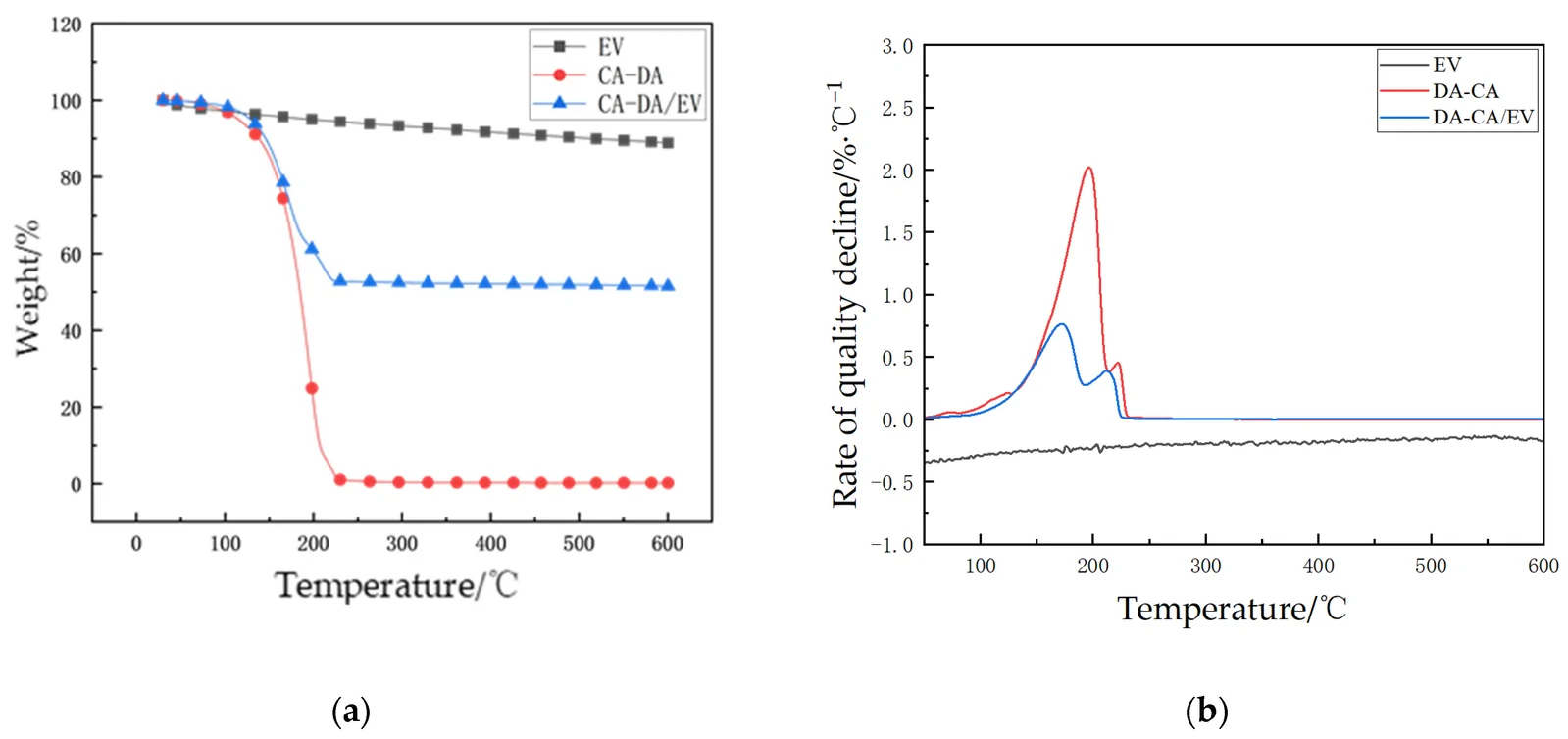
(**a**) Thermogravimetric curves; (**b**) derivative thermogravimetric curves.

Figure 11 shows the thermogravimetric (TG) and derivative thermogravimetric (DTG) curves of EV, DA-CA, and DA-CA/EV. As the temperature increased, the mass of all samples gradually decreased and eventually stabilized. Over the range of 0–600 °C, EV exhibited a mass loss of approximately 15.8%, mainly due to the volatilization of a small amount of internal moisture. No abrupt mass change was observed during the test, indicating that EV did not undergo significant thermal decomposition and therefore possessed good thermal stability. The DA-CA eutectic PCM exhibited an initial decomposition temperature of approximately 133.2 °C, and its main mass-loss process ended at approximately 222.6 °C. In contrast, DA-CA/EV showed an initial decomposition temperature of approximately 134.3 °C, with mass loss ending at 228.4 °C. The initial decomposition temperatures of both materials were much higher than their expected service temperature range, which is typically below 100 °C, confirming their reliable thermal stability under practical application conditions.

The final mass loss of DA-CA reached 99.83%, indicating nearly complete decomposition. By contrast, the final mass loss of DA-CA/EV was 49.52%, which is consistent with the effective adsorption and form-stabilization effect of EV on DA-CA during composite preparation.

### 3.8. Thermal Conductivity Analysis

**Figure 12:**
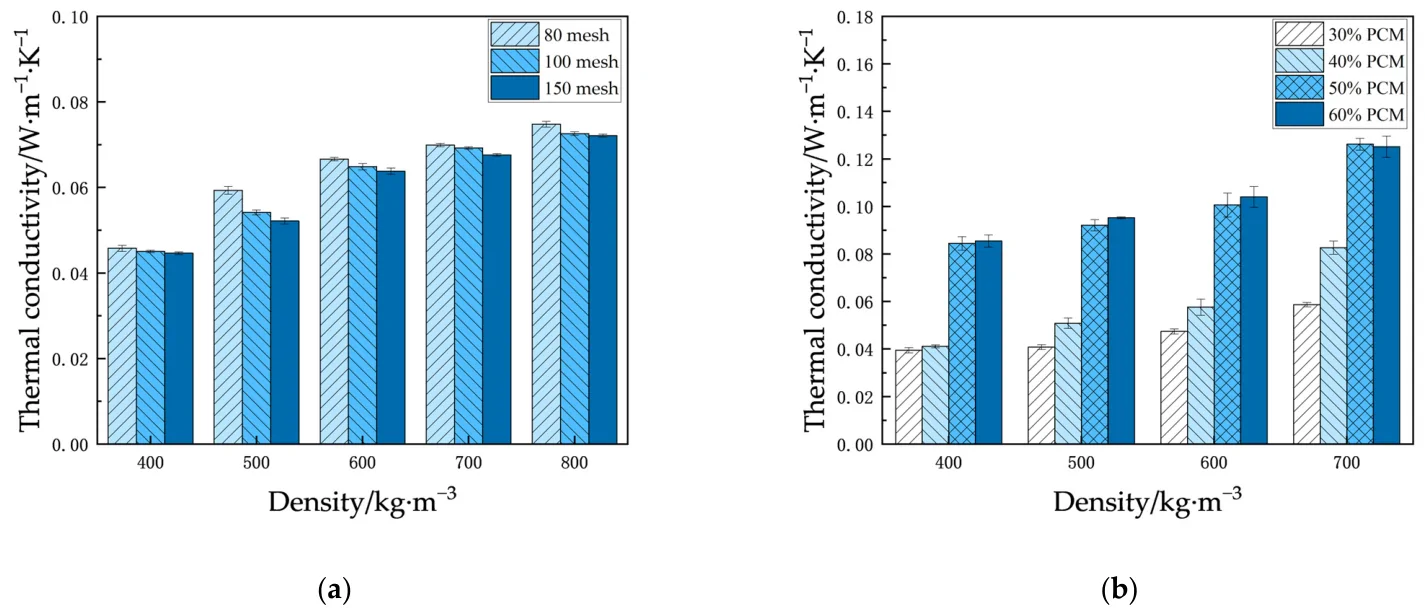
Thermal conductivity of DA-CA/EV: (**a**) EV with different particle sizes; (**b**) PCM adsorption at different mass contents.

Figure 12a shows that the thermal conductivity of the composite PCM increased with its density. Higher density reduces internal porosity and provides a more continuous and less obstructed heat-conduction pathway within the solid. In addition, because 80-mesh particles are larger, the number of particle interfaces within the same volume is much smaller than that in 150-mesh particles. Consequently, fewer interfacial barriers are encountered along the heat-transfer pathway, allowing heat flux to pass more readily and resulting in higher macroscopic thermal conductivity. Thermal conductivity was also measured under different density conditions after the addition of 30%, 40%, 50%, and 60% PCM to 100-mesh EV.

As shown in Figure 12b, the overall tendency for thermal conductivity to increase with density remained unchanged. When the PCM content reached 50%, the pores in EV were essentially filled with PCM, and the internal air content was substantially reduced, resulting in a marked increase in thermal conductivity. Once the thermal conduction network was established, further increasing the PCM content to 60% did not create additional heat-transfer pathways. At this stage, the thermal resistance was mainly limited by the intrinsic thermal conductivity of the PCM, leading to a plateau in the composite’s thermal conductivity. Therefore, using 100-mesh EV with approximately 50% PCM adsorption while maintaining a high density provides the optimal material performance. Nevertheless, the thermal conductivity of the composite system remains limited. Future studies could improve its thermal performance by incorporating thermally conductive fillers such as graphite or carbon fibers.

### 3.9. Experimental Investigation of PCM Hydraulic Concrete

**Figure 13:**
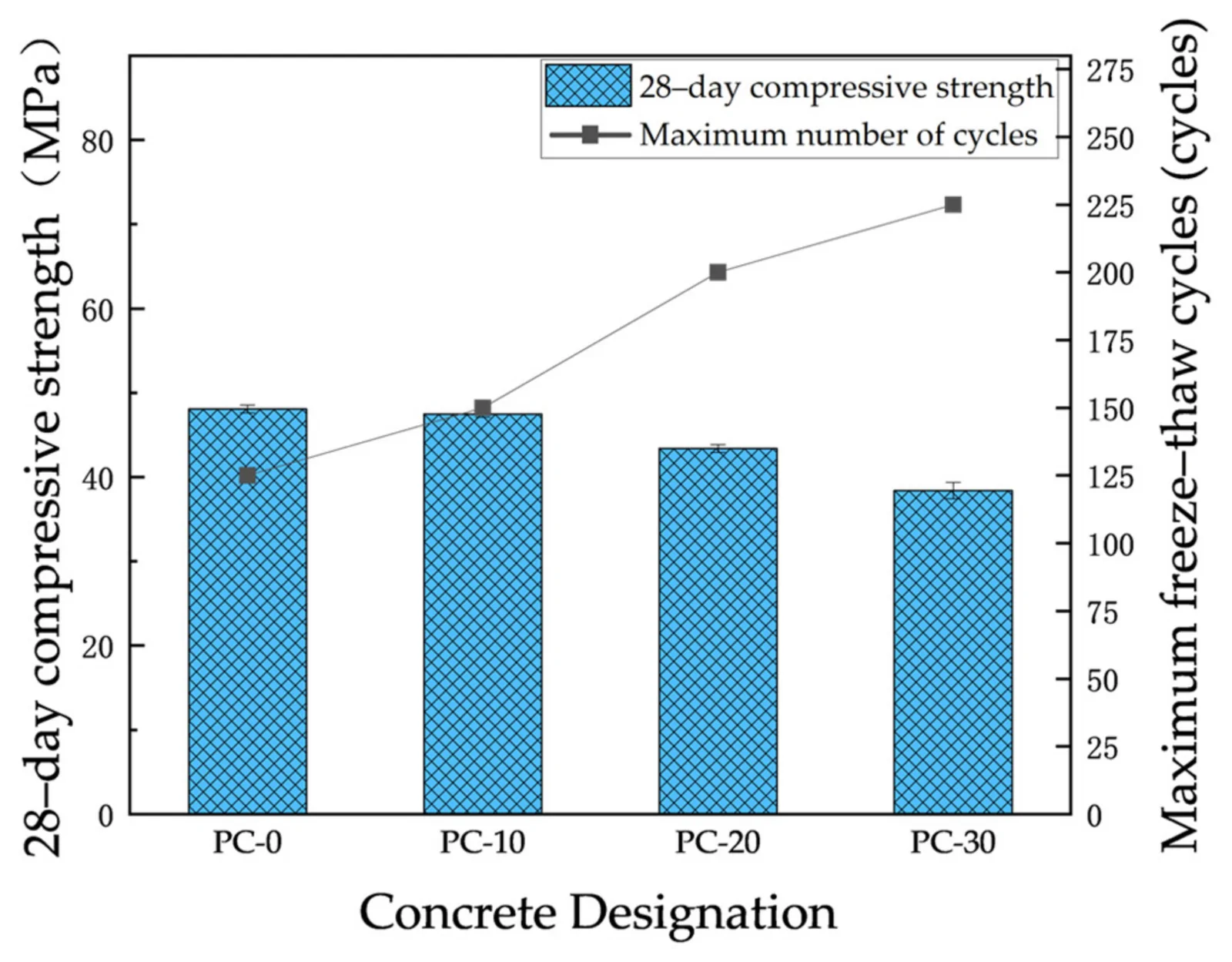
Effect of PCM content on durability and compressive strength of hydraulic concrete.

As shown in Figure 13, the incorporation of phase-change aggregates led to a reduction in the compressive strength of concrete, and the strength loss became more pronounced with increasing aggregate content. At the age of 28 days, the compressive strength of the PC-0 mixture was 48.1 MPa. As the dosage of phase-change aggregates increased, the 28-day compressive strength decreased successively to 47.3 MPa, 43.5 MPa, and 38.8 MPa. Compared with PC-0, the PC-30 mixture exhibited a reduction of approximately 19.33% in its 28-day compressive strength. These results indicate that the decrease in compressive strength was approximately linearly correlated with the content of phase-change aggregates. This strength reduction can be mainly attributed to the interfacial transition zone between the phase-change aggregates and the cement paste, which generally acts as a weak region in concrete. In addition, the intrinsic strength of expanded vermiculite is significantly lower than that of natural aggregates, making it more susceptible to stress concentration and thereby reducing the overall mechanical strength of the concrete. Nevertheless, all phase-change concrete mixtures still satisfied the requirements for relatively high strength grades at 28 days.

As the PCM content increased from 0% to 30%, the ultimate number of freeze–thaw cycles sustained by the concrete increased continuously from 125 to 225, showing a strong positive correlation between the PCM dosage and freeze–thaw resistance. This result suggests that the incorporation of phase-change materials can effectively improve the freeze–thaw durability of concrete. Moreover, the frost-resistance limit increased monotonically with the PCM content, without any turning point or decline in performance, indicating that the beneficial regulatory effect of PCM remained dominant within the investigated dosage range. Before the PCM content reached 20%, increasing the PCM dosage resulted in an accelerated improvement in freeze–thaw resistance. However, when the PCM content exceeded 20%, the additional benefit became markedly reduced. This phenomenon can be explained from two aspects. First, when the PCM content reached approximately 20%, the latent-heat-based thermal regulation capacity inside the concrete had been sufficiently activated, and further PCM addition produced a diminishing marginal effect in suppressing frost-heaving damage. Second, an excessively high PCM content may weaken the interfacial bonding within the concrete matrix and increase harmful porosity. These adverse effects on mechanical properties and pore structure may partially offset the freeze–thaw resistance enhancement derived from thermal regulation.

## 4. Conclusions

In this study, a DA-CA binary eutectic PCM was prepared by melt blending for potential application in improving the freeze–thaw resistance of hydraulic concrete in dam engineering. EV was used to encapsulate and form-stabilize the eutectic PCM. The phase-transition temperature, latent heat, chemical structure, mass stability, and thermal conductivity of the composite were characterized. The main conclusions are as follows:

(1) The eutectic mass ratio of DA to CA was 35:65. After its adsorption into EV, DSC measurements showed that the solidification phase-transition temperature and latent heat of the composite were 2.37 °C and 120.8 J/g, respectively.

(2) SEM analysis showed that EV can stably adsorb the PCM through capillary forces and surface tension, forming a structurally stable composite phase-change thermal storage system. When 100-mesh EV was used as the support matrix, no leakage was observed in the DA-CA/EV composite PCM. In addition, optimal thermal conductivity can be achieved by adsorbing approximately 50% of the phase-change material (PCM) while maintaining a high packing density.

(3) FT-IR analysis indicated that DA, CA, and EV were combined through physical adsorption without chemical reaction. The initial decomposition temperature of DA-CA/EV was 134.3 °C. After 300 thermal cycles, both the mass and the phase-transition temperature of the material showed no obvious variation, demonstrating the excellent thermal stability and reliability of DA-CA/EV.

(4) Incorporating phase-change aggregates linearly reduced concrete’s compressive strength yet steadily boosted its freeze–thaw resistance, and all mixtures still satisfied high strength grade demands despite slowed frost-resistance improvement beyond a 20% PCM content. From the perspective of intrinsic material properties, the prepared composite has potential for application in hydraulic concrete engineering. Further systematic concrete tests are required to evaluate its practical engineering performance.

## Figures and Tables

**Table 1:** Effects of EV particle size on PCM adsorption efficiency.

Particle Size (mesh)	Mass of DA-CA (g)	Total Mass After Adsorption (g)	Average Adsorption Rate (%)
40	5	Unformed	——
40	5	Unformed	
80	5	9.49	53.49
80	5	9.21	
100	5	9.43	53.72
100	5	9.19	
150	5	9.14	54.32
150	5	9.27	

**Table 2:** Table of criteria for evaluating leachability stability.

Leakage Category	Leakage Percentage, φ (%)	Stability
Minimal leakage (considered no leakage)	φ ≤ 10	Highly stable
Trace leakage	10 < φ ≤ 15	Stable
Slight leakage	15 < φ ≤ 30	Generally stable
Moderate leakage	30 < φ ≤ 50	Unstable
Severe leakage	φ > 50	Extremely unstable

**Table 3:** The thermal properties and adsorption rates of the PCM compared with results reported in the literature.

Materials	Solidification Phase-Change Temperature (°C)	Latent Heat (J/g)	Adsorption Rate (%)	Reference
n-tetradecane/epoxy resin	6	112	40	[35]
melamine–urea/formaldehyde	−4.21	15.41	81.99	[36]
n-tetradecane–n-hexadecane/ modified graphite	1.3	76.71	98	[37]
polyethylene glycol 600/silica	9	66.24	61	[38]
n-tetradecane/EP	5.51	110.9	83.33	[39]
DA-CA/EV	2.37	113	53.72	This work

**Table 4:** Mass change of DA-CA/EV composite PCM after thermal cycling.

Thermal Cycles	Mass, g	Mass Loss Rate, %
1	0.688	0
100	0.682	0.87
200	0.681	1.02
300	0.681	1.02

## Data Availability

The original contributions presented in this study are included in the article. Further inquiries can be directed to the corresponding author.

## Abbreviations

The following abbreviations are used in this manuscript:

DA	n-Decanol
CA	n-Octanoic acid
DA-CA	n-Decanol/n-octanoic acid binary eutectic mixture
EV	Expanded vermiculite
DA-CA/EV	Form-stable composite phase-change material prepared by adsorbing DA-CA into expanded vermiculite
PCM	Phase-change material
PC-i	Concrete with PCM dosages of i% (i = 0, 10, 20, 30)
